# Nonmalignant tracheal stenosis: presentation, management and outcome in limited resources setting

**DOI:** 10.1186/s13019-024-02480-w

**Published:** 2024-01-23

**Authors:** Dereje Gulilat, Abraham Genetu, Segni Kejela, Seyoum Kassa, Abebe Bekele, Ayalew Tizazu

**Affiliations:** 1 Department of Surgery, School of Medicine, College of Health Sciences, Addis Ababa University, Addis Ababa, Ethiopia; 2 University of Global Health Equity, Kigali, Rwanda

**Keywords:** Tracheal stenosis, Tracheal resection, Bronchoscopic dilation

## Abstract

**Background:**

Nonmalignant tracheal stenosis is a potentially life threatening conditions that develops as fibrotic healing from intubation, tracheostomy, caustic injury or chronic infection processes like tuberculosis. This is a report of our experience of its management with tracheostomy, rigid bronchoscopic dilation and surgery.

**Methods:**

Retrospective study design was used. 60 patients treated over five years period were included.

**Results:**

Mean age was 26.9 ± 10.0 with a range of 10–55 years. Majority (56 patients (93.3%)) had previous intubation as a cause for tracheal stenosis. Mean duration of intubation was 13.8 days (range from 2 to 27 days). All patients were evaluated with neck and chest CT (Computed Tomography) scan. Majority of the stenosis was in the upper third trachea − 81.7%. Mean internal diameter of narrowest part was 5.5 ± 2.5 mm, and mean length of stenosed segment was 16.9 ± 8 mm. Tracheal resection and end to end anastomosis (REEA) was the most common initial modality of treatment followed by bronchoscopic dilation (BD) and primary tracheostomy (PT). The narrowest internal diameter of the tracheal stenosis (TS) for each initial treatment category group was 4.4 ± 4.3 mm, 5.1 ± 1.9 mm and 6.7 ± 1.6 mm for PT, tracheal REEA and BD respectively, and the mean difference achieved statistical significance, F (10,49) = 2.25, *p* = 0.03. Surgery resulted in better outcome than bronchoscopic dilation (89.1% vs. 75.0%).

**Discussion and conclusion:**

Nonmalignant tracheal stenosis mostly develops after previous prolonged intubation. Surgical resection and anastomosis offers the best outcome.

## Introduction

Nonmalignant tracheal stenosis develops as a result of fibrotic healing from prolonged intubation, tracheostomy, caustic injury or chronic infection processes like tuberculosis [1–4]. The most common cause, postintubation tracheal stenosis (PITS), is narrowing of tracheal lumen resulting from cicatricial healing of a transmural airway injury from prolonged intubation [5, 6]. Tracheal ischemia results mainly from high volume-high pressure cuffs that results in fibrotic healing or tracheoesophageal fistula in some cases [7, 8]. Despite the advent of low pressure high volume cuff, PITS still remains a challenging problem [4]. Reported prevalence of stenosis related to prolonged intubation ranges from 0.6 to 21% [9].

Symptoms develop gradually, usually presenting 1 to 6 weeks after extubation [9]. Patients usually present with progressive shortness of breath, stridor, cough, wheezing and recurrent pneumonia [10]. While definitive management remains to be surgical resection and anastomosis of trachea, conservative options with bronchoscopic dilation are options of initial treatment or as definitive treatment for surgically unfit patients [4, 10].

With increased surgical volume and intensive care facilities in Ethiopia, the number of patients who present with tracheal stenosis could gradually increase. In resource limited settings like Ethiopia where bronchoscopic dilations are not widely available, surgical treatment remains the most widely used mode of treatment. In this paper, we describe our experience in patients with nonmalignant tracheal stenosis, describing the pattern of presentation, diagnosis, management and outcome.

## Methods

Study design: Retrospective cross sectional study design was used.

Study setting: This is a report of patients operated at the Tikur Anbessa Teaching Hospital, Menelik II Hospital and St Peter Referral Hospital, which are all in Addis Ababa, Ethiopia.

Study period: Data was retrospectively collected from patients treated over five years period from March 2018 up to March 2023. Patients treated for tracheal stenosis that resulted from nonmalignant causes were identified from admission and operative logbooks.

### Data collection

Data were collected using a structured data collection tool. All patients’ charts were used to collect data on demographics, symptoms at presentation, history, physical examination findings, treatment and progress. CT scans were examined to determine grade, length and site of stenosis. During the study period, 65 patients were treated, all were included, except for 5 patients whose medical charts could not be retrieved.

### Data analysis

Data were analyzed by SPSS statistical software version 25. Descriptive statistics was used to summarize the study data. Inferential statistics were used to analyze treatment outcomes with regard to independent variables and treatment offered.

### Ethical consideration

The study was approved by Research Committee of the Department of Surgery, College of Health Sciences, Addis Ababa University (RC/DOS/132/2022). Patients signed informed consent when data was collected during their follow up, and verbal consent was taken when they were interviewed over the phone.

### Patient evaluation

When patients presented with symptoms of stridor or difficulty of breathing after previous history of intubation, tracheostomy or tracheal surgery, they were evaluated with history, physical exam and 128 slice CT scan of the neck and chest. CT scan was used for grading and surgical approach planning. Currently, treatment options available in the country for these patients are tracheostomy, bronchoscopic dilation and surgery.

Based on neck and chest CT scan, degree of stenosis and location were described as was suggested by Freitag et al [11]. The grading of stenosis is as follows.

- Grade 0 – No appreciable stenosis.
- Grade 1 – up to 25% stenosis.
- Grade 2–25–50% stenosis.
- Grade 3 − 50–75% stenosis.
- Grade 4–75–90% stenosis.
- Grade 5 – Complete obstruction.

Location of stenosis is reported as;

I. Upper third of the trachea.
II. Middle third of the trachea.
III. Lower third of the trachea.
IV. Right main bronchus.
V. Left main bronchus.

Treatment options and algorithm.

Tracheostomy was the only available option for a long time. Over last few decades, thoracic surgeons have been doing surgical REEA. Recently, bronchoscopic dilation is done by one surgeon in one of the treating hospitals (Tikur Anbessa Specialized Hospital). Currently, REEA and bronchoscopic dilation are available only in Addis Ababa, Ethiopia. Other treatment options like stenting, laser or cryotherapy are not available in the country. Therefore, we do not strictly follow algorithms suggested from well equipped settings. Hence, a patient who is a candidate for surgery but is located far away from Addis Ababa could have tracheostomy placed and then referred to our centers. Once patients arrived to our centers, they are evaluated with CT and/or bronchoscopy. Those with simple stenosis (< 1 cm, granuloma), mild stridor after surgery, or those not fit for surgery are generally offered bronchoscopic dilation. Complex stenosis is managed with tracheal REEA.

Tracheostomy.

Tracheostomy was used in different scenarios for our patients.

We used the following operational definitions;

- Tracheostomy after Prolonged intubation (TAPI): These are tracheostomy that were done following prolonged intubation during the first admission. These patients are excluded when analyzing symptoms on presentation, as they are already on tracheostomy before they developed obstructive symptoms.
- Tracheostomy as Initial treatment (TAIT): These are tracheostomy that were done to patients that present with symptoms of upper airway obstruction. When tracheostomy was done for our patients before bronchoscopic dilation or surgical resection of trachea, we considered it as the *initial treatment*. This often happens because definitive treatment is available only in the capital city, Addis Ababa, hence, keeping patients stable before referral to these centers.
- Protective Tracheostomy: this is when tracheostomy was done during the tracheal resection/repair procedure. The role of tracheostomy is this case is to protect the tracheal anastomosis.
- Permanent tracheostomy: this is done in patient with failed definitive care (dilation and/or surgery).

Bronchoscopic dilation.

When stenosis is grade ≤ 4 and is a short segment, or when it involves multi-level segment of trachea/bronchi, the first line treatment we offered was bronchoscopic dilation. Dilation is done under sedation and short acting muscle relaxants. Rigid bronchoscopy (Karl Storz, Germany) is inserted, the lumen is intubated with the balloon (CRE™ Pulmonary Balloon Dilatation Catheter, Boston Scientific, USA) under direct vision. Once the balloon is at the site of obstruction, we kept the cuff dilated at 3 atmosphere (atm) pressure by pushing saline in to the attached syringe with pressure manometer for 30–120 s, which can result in 12 mm diameter. This might be repeated depending on response. Dexamethasone 4 mg intramuscular (IM) twice daily is initiated and patients are followed. If stridor is still present, or recurs in few days, repeat session of dilations are done in a week time from initial session. If good response is not observed, and resection is possible, the next step is surgical resection and anastomosis.

### Surgery and airway management perioperatively

**Fig. 1:**
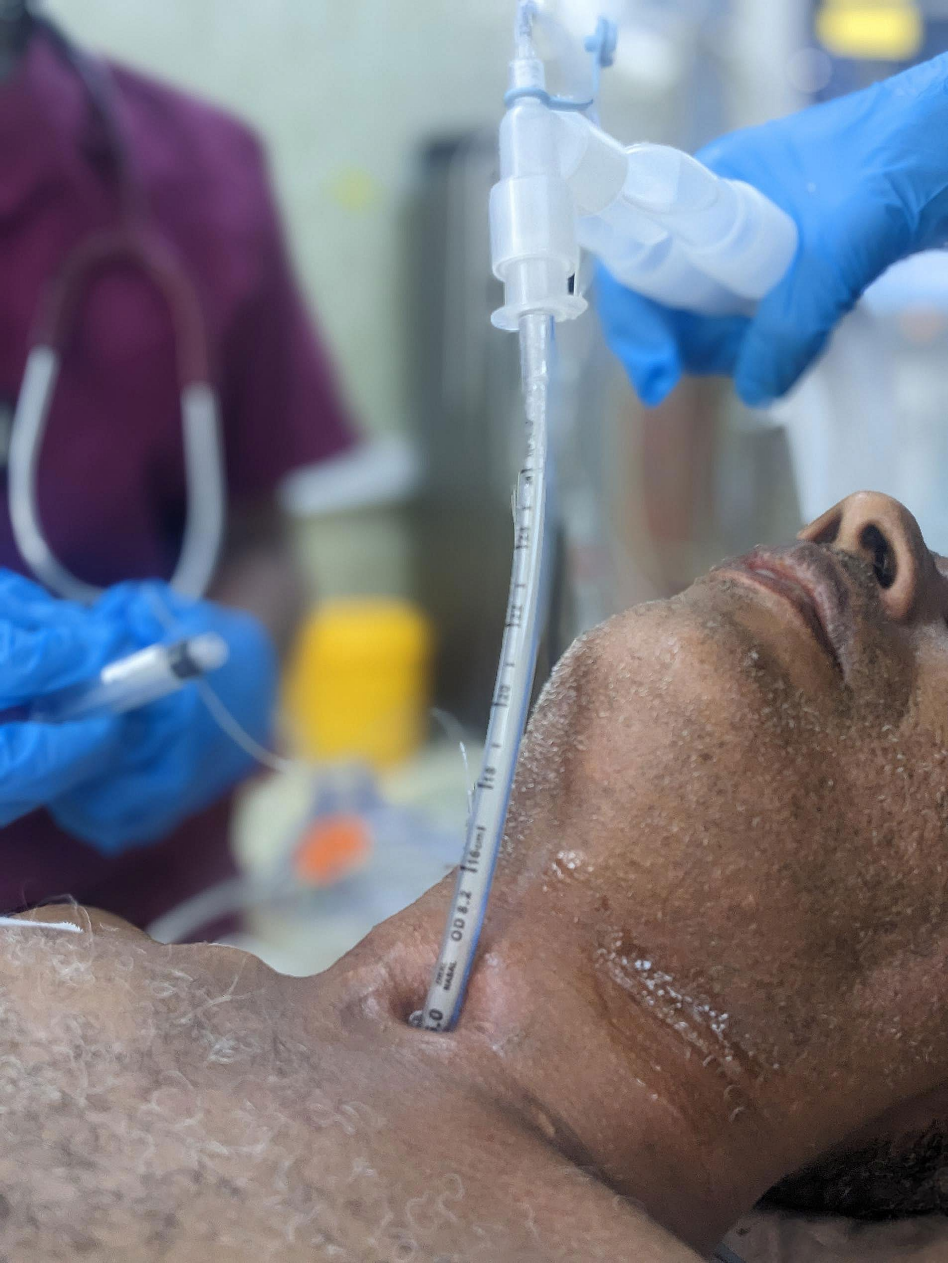
Patient is intubated via tracheostomy site before the surgical field is prepared with antiseptics

**Fig. 2:**
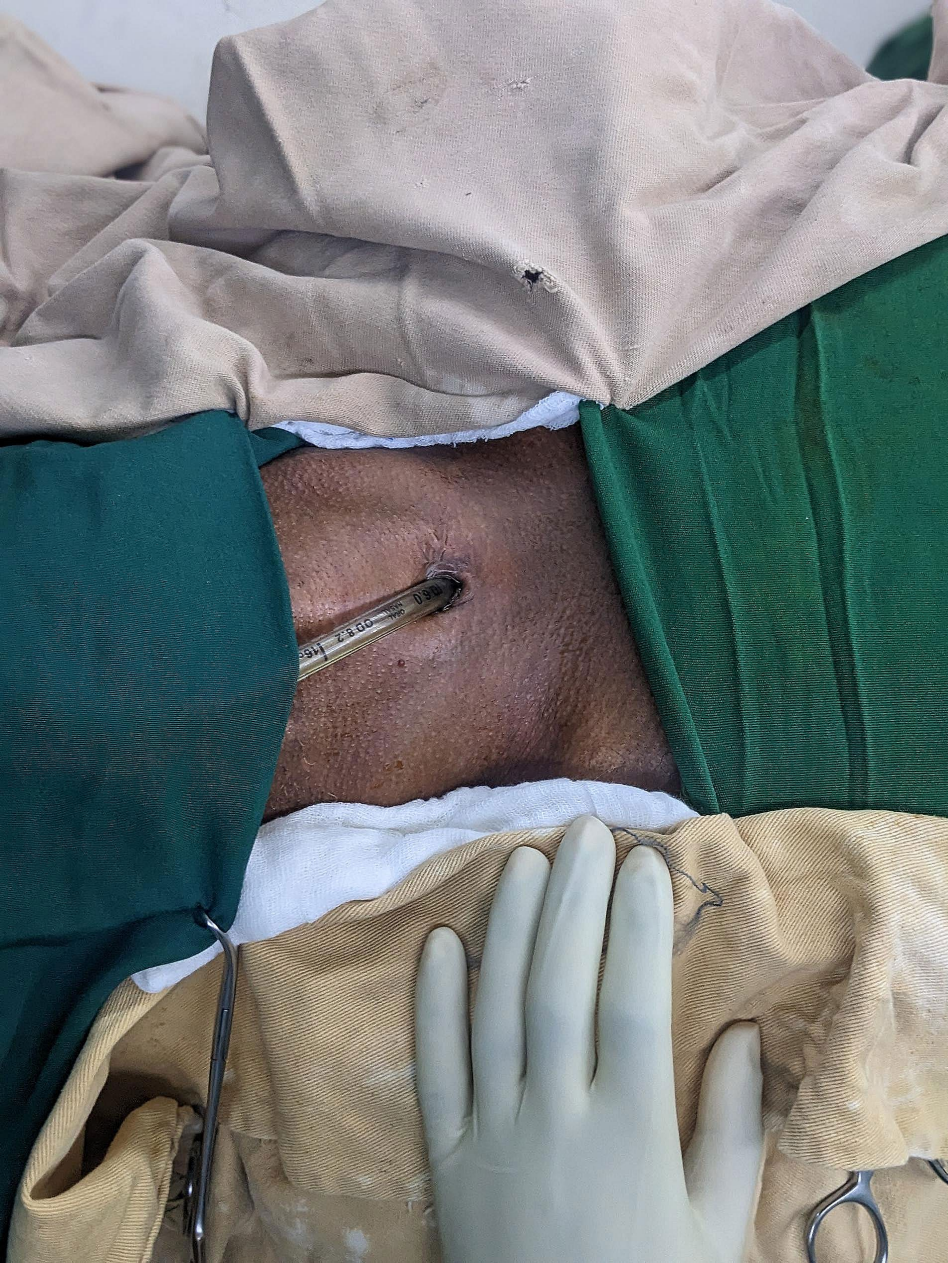
Once field is prepared, surgeon intubates through tracheostomy site with sterile endotracheal tube and the tube is passed below drapes to the anesthesia machine side. N.B. Patient’s head is to the left of the figure

Collar incision was performed for most patients, as the stenosis was in upper or middle third of trachea. Patrial sternotomy was used when adequate mobilization was not achieved. When the patient is already on tracheostomy, intubation is first done with endotracheal tube (ETT) thru tracheostomy site. After draping, another sterile tube is used to intubate, and it is extended with fitting sterile chest tube to give adequate length to reach for cross field ventilation. The thyroid is often divided along midline and retracted laterally. Stenosed trachea is then dissected circumferentially after which it is resected. Posterior interrupted sutures are placed with prolene 3/0. Another endotracheal tube is now passed through the mouth, through the anastomosis down to trachea and ventilation is done via this new tube. Anterior sutures are placed and anastomosis is completed. Anastomosis is checked for air leak. Patients are often extubated on table. See Figs. 1 and 2.

If the patient is not on tracheostomy, usual intubation was tried with small size ETT for less severe stenosis. If the ETT cannot pass below stenotic site, it was be kept above it and ventilation is continued. Once trachea is opened, the distal trachea is intubated and ventilation continued. The usual dissection, resection and anastomosis is done.

Outcome of treatments was judged with the same grading system used by Grillo et al [7].

1. Good results were characterized by the ability to perform usual activities and x/ray or bronchoscopy that showed good airway patency.
2. Satisfactory results mean patients had no issues with normal activities but were somewhat stressed with exercise or had vocal cord dysfunction or evidence of narrowing by x/ray or bronchoscopy.
3. Failure was defined as need for permanent tracheostomy.

## Results

### Results

#### Patient profile, causes of stenosis and presentation

Over the 5 years period, 60 patients were treated for nonmalignant tracheal stenosis (TS). The mean age was 26.9 ± 10.0 with a range of 10–55 years. Majority (56 patients (93.3%)) had previous intubation as a cause for tracheal stenosis. Mean duration of intubation was 13.8 days (range from 2 to 27 days). Most common reasons for intubation were traumatic brain injury (*n =* 20, 35.7%) and respiratory failure due to Acute Respiratoy Distress Syndrome (ARDS) or sepsis (*n =* 10, 17.9%). Presenting symptoms were stridor, shortness of breath and exercise intolerance. Participants’ clinical and demographic values are shown in Table 1.

**Table 1:** Clinical characteristics of patients with tracheal stenosis (*n* = *60*)

Sex	Male	46 (76.7%)
	Female	14 (23.3%)
Comorbidities	Diabetes mellitus	2 (3.3%)
	HIV	2 (3.3%)
	Tuberculosis	2 (3.3%)
	Immunosuppressive disease on steroids	1 (1.7%)
Cause of stenosis	Post intubation	56 (93.3%)
	Neck injury	2 (3.3%)
	Caustic injury	1 (1.7%)
	Tuberculosis	1 (1.7%)
Reason for previous intubation	Traumatic Brain Injury	20 (35.7)
	ARDS or Sepsis	10 (17.8%)
	Suicidal attempt	5 (9.9%)
	Burn	3 (5.4%)
	After surgery	3 (5.4%)
	GBS	3 (5.4%)
	Stroke	2 (3.6%)
	Complicated COVID 19	2 (3.6%)
	Generalized tetanus	2 (3.6%)
	Status epilepticus	2 (3.6%)
	Eclampsia	2 (3.6%)
	Medical coma	2 (3.6%)
	Meningitis	1 (1.8%)
Tracheoesophageal fistula	Present	4 (6.7%)
	Absent	56 (93.3%)
Grade of stenosis	Grade 1	0(0.0)
	Grade 2	7 (11.7)
	Grade 3	24 (40.0)
	Grade 4	24 (40.0)
	Grade 5	5(8.3)
Location	Upper third of trachea	49 (81.7)
	Mid third of trachea	10(16.7)
	Lower third of trachea	1(1.7) *
	Right main bronchus	1(1.7) *
	Left main bronchus	0(0.0)

* One patient had a caustic injury that involved lower third of trachea and right main bronchus

*HIV: Human Immunodeficiency Virus; GBS: Guillain-Barré syndrome*

### Diagnosis and initial management

All patients had neck and chest CT scan done. Majority (81.7%) had stenosis in the upper third trachea. Severity of stenosis was grade 3 and 4 in 40% each, while 8.3% had complete/near complete obstruction. Mean internal diameter of narrowest part was 5.5 ± 2.5 mm, and mean length of stenosed segment was 16.9 ± 8 mm. Associated tracheoesophageal fistula (TEF) was detected in 6.7% of the studied patients. The mean durations of intubation for those with TEF and without were 18.7 ± 6.1 and 13.6 ± 5.9 days, respectively. The difference in the mean values was not statistically significant, F(1,54) = 2.06, *p* = 0.157. (see Table 2).

**Fig. 3:**
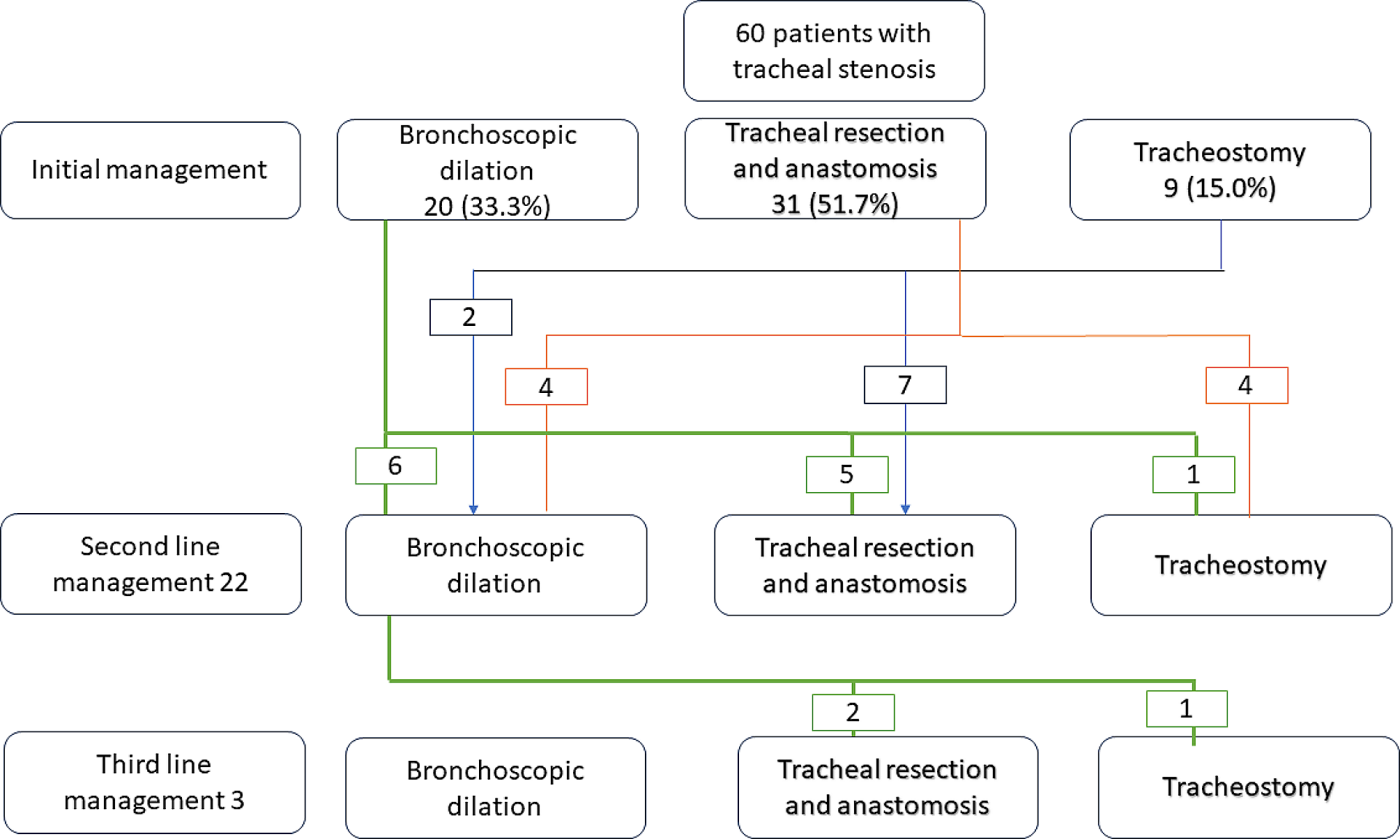
Flow diagram showing initial, second line and third line management

Bronchoscopic dilation, tracheal resection and/or tracheostomy were used as management options utilized. (See flow diagram on Fig. 3.) Tracheal resection and end to end anastomosis (REEA) was the most common initial modality of treatment followed by bronchoscopic dilation (BD). The mean lengths of TS among patients treated with bronchoscopic dilation, primary tracheostomy (PT) and tracheal REEA were 14.05 ± 8.04 mm, 17.6 ± 9.5 mm and 18.6 ± 7.2 mm respectively. The mean difference was not statistically significant, F (21,38) = 1.38, *p* = 0.188. The narrowest internal diameter of the TS for each initial treatment category group was 4.4 ± 4.3 mm, 5.1 ± 1.9 mm and 6.7 ± 1.6 mm for PT, tracheal REEA and BD respectively, and the mean difference achieved statistical significance. F(10,49) = 2.25, *p* = 0.03.

**Table 2:** CT characteristics of stenosed tracheal segment in relation to initial treatment categories

	Initial treatment category		
	Bronchoscopic dilation	Surgery	Tracheostomy
Mean internal diameter in mm	6.73 ± 1.65	5.1 ± 1.95	4.44 ± 2.45
Mean length in mm	14.05 ± 8.03	18.61 ± 7.23	17.56 ± 9.55
Grade 4 or grade 5 stenosis	15%	64.5%	66.6%

Bronchoscopic dilation was done for 26 (43.3%) patients. It was used as initial treatment for 20 (33.3%), as second line management after REEA or initial tracheostomy for 6 (10.0%). When used alone, BD resulted in good or satisfactory outcome in 75%. Overall, 13 (50.0%) had good outcome, 12 (46.2%) had satisfactory outcome, and 1 (3.8%) failed. Majority (63%) required one time dilation; the rest had 2–8 sessions. The one patient who failed to respond was operated and was rendered symptom free. There were no complications from bronchoscopic dilation.

46 patients (76.7%) had tracheal resection and end to end anastomosis. REEA was used as initial management for 31 patients (51.7%). All were operated via cervical collar incision, and one patient required an additional partial sternotomy. Mean length of trachea resected was 2.4 ± 0.6 cm. Maximum length of resection was 3.5 cm. 4 patients had associated tracheoesophageal fistula which was repaired during resection. All except 3 (6.5%) were extubated immediately. All were followed in intensive care unit for one to three days. All patients who underwent dilation or surgery were given dexamethasone 4 mg IM twice daily for 5 days. Overall, 41 (89.1%) had good or satisfactory outcome. Some degree of stridor or distress during activities was observed in 4 (8.6%) which is judged as satisfactory outcome. Rigid bronchoscopy showed granulation tissue at anastomotic site and dilation resulted in good outcome. 5 (10.9%) had failure, which were subsequently managed with permanent tracheostomy.

There were postoperative complications in 6 (13.0%) patients; pneumothorax in 4 (8.7%), subcutaneous emphysema in 1(2.2%) and small anastomotic dehiscence in 1 (2.2%). There was no postoperative voice change, however, two patient who had bullet injury had already recurrent laryngeal nerve before surgery.

Tracheostomy was done for some patients who had prolonged intubation, as prophylaxis to prevent post-intubation tracheal stenosis, as initial treatment when patients presented with symptoms of tracheal stenosis (defined as ‘initial management’) or when dilation/surgery failed as permanent tracheostomy. Tracheostomy was used at some time in the course of treatment for 23 (38.3%). It was done mainly for prolonged intubation (39.1%) and as initial management (39.1%).

Four patients had tracheoesophageal fistula (TEF) in addition to tracheal stenosis. Three had PITS and one had zone II neck injury from bullet. Esophageal fistula repair with tracheal REEA was done for 3 of them with good outcome. For one patient, TEF segment was long prohibiting tracheal REEA, hence, fistula was repaired and permanent tracheostomy was placed.

### Outcome of treatment and follow up

Mean duration of follow up/time from treatment was 25.6 months (range: 1–50 months). 80% of the patients were followed for at-least one year while 53.3% were followed for at-least 2 years After a combined surgical and bronchoscopic dilation, good and satisfactory results were achieved in 55 patients (91.7%). There was nomortality.

**Fig. 4:**
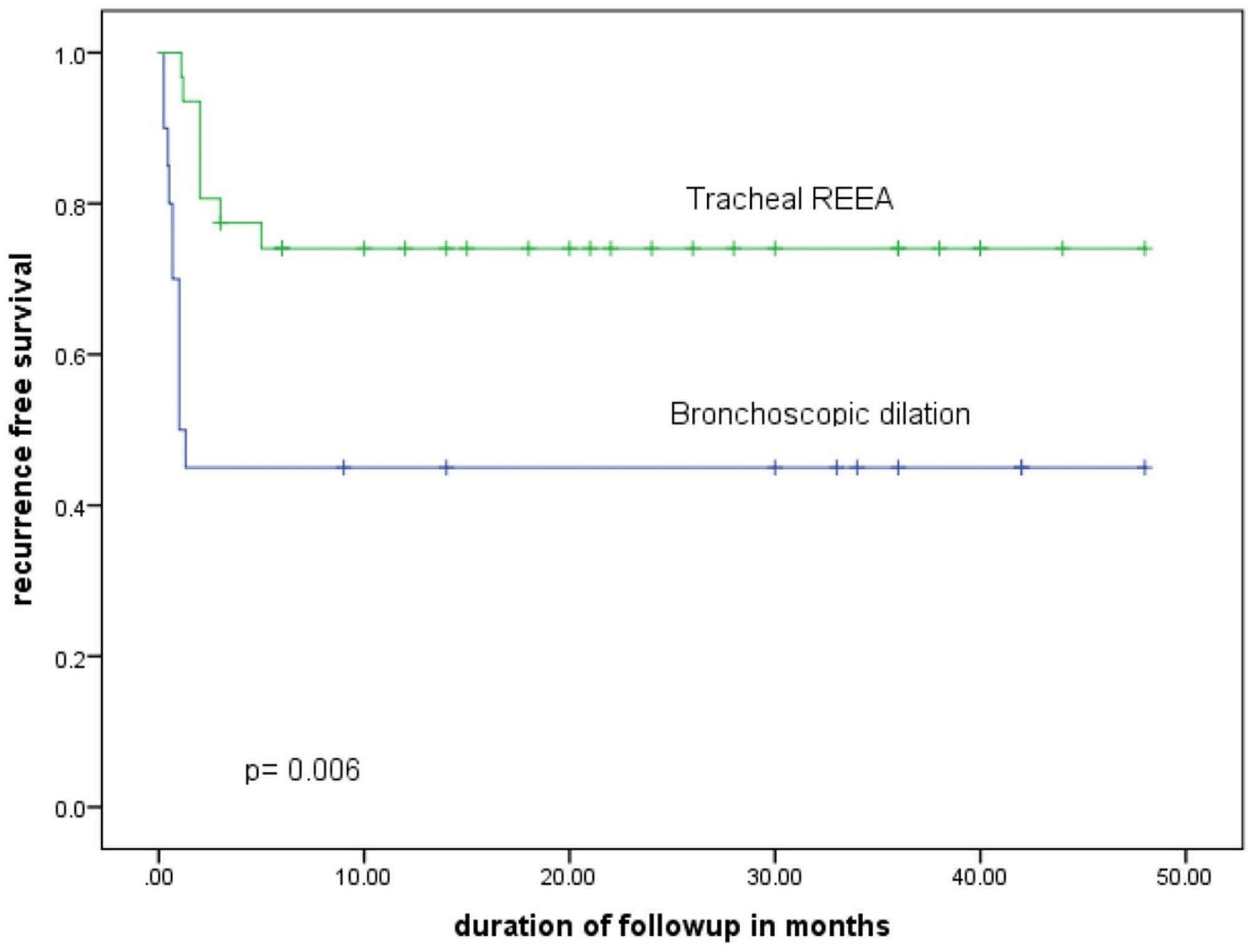
Kaplan–Meier Estimates of recurrence free Survival

After the initial intervention, 55% of BD patients required one or more interventions while 25.8% tracheal REEA required reintervention for recurrence. All patients with initial tracheostomy underwent either BD (in 2 of the 9 patients) or tracheal REEA (in 7 of the 9 patients). All initial tracheostomy procedures were regarded as temporizing measures since all patients required re-interventions with the other modalities, and so excluded from cox analysis. Compared to BD, tracheal REEA patients had higher recurrence free survival after the initial intervention (hazard ratio for recurrence 0.15; 95% confidence interval 0.049–0.488; *p* = 0.006). In a multivariable logistic regression, besides initial treatment, recurrence was associated with the length of the TS (adjusted odds ratio 1.29; 95% confidence interval 1.06–1.56; *p* = 0.01). (See Fig. 4).

## Discussion

Tracheal stenosis in our study mostly happens after previous prolonged intubation. Traumatic brain injury was the most common reason for prolonged intubation. Surgical resection and bronchoscopic dilation resulted in good or satisfactory outcome in 91.7%. In this study of patients with non-malignant tracheal stenosis of unmatched groups with regards to the diameter of the narrowest segment, even though the initial success rate favored tracheal REEA compared to BD, the end of therapy patient symptom related response was equivalent when the patients were allowed to crossover to surgical intervention. In a group treated with only BD the satisfactory symptomatic improvement rate was 75% compared to close to 90% in tracheal REEA group. This signifies that, when patients are carefully selected and cases with symptomatic recurrence were allowed to crossover to surgical interventions, both BD and tracheal REEA can achieve high rates of satisfactory outcomes.

Although endotracheal intubation is an important element of critical care, it is associated with complications like tracheal stenosis, tracheoesophageal fistula or tracheoarterial fistula [12]. Incidence of postintubation tracheal stenosis (PITS) is estimated to be at 1%, however, use of low pressure high volume cuffs has reduced the incidence dramatically [5]. When the cuff pressure is > 30 mmHg, it could result in ischemia followed by fibrosis and progressive stenosis [13]. The pressure estimation technique using manual compression is found to be insensitive in predicting high cuff pressure, so routine pressure monitoring and low pressure maintenance is generally recommended [14, 15]. For such reason, routinely monitor intracuff pressure and maintain it below 30 mm Hg, or as low a level as needed to create an adequate seal for ventilation is recommended by the American Association for Respiratory Care [14]. The risk of PITS is also increased with prolonged intubation, which is arbitrarily defined as intubation for more than 7 days [15]. However, PITS has been reported even after brief intubation of less than 24 h [16]. Some reports, similar to ours, found TS at a mean duration of intubation of 14 days [9]. Because of risk of PITS, tracheostomy is usually done when patients stay intubated for prolonged days. However, while some evidences showed that early tracheostomy can lead somewhat lowered mortality rate and pneumonia diagnosis, but its effect on TS is largely lacking [17].

The usual presenting symptoms are shortness of breath and stridor which start after 1–6 weeks of extubation in most reports. The tracheal diameter is less than 50% when these symptoms appear [18]. In cases of post intubation stenosis, usually symptoms start after one week. Our patients started to have symptoms after mean of 13.8 days, however, there was one patient who became symptomatic on day of extubation after he remained intubated for 22 days, while some stayed up to 90 days showing variable degree of progression.

Evaluation of location, severity and length of stenosis is best performed with combination of bronchoscopy and CT scan. In one study that evaluated the usefulness and accuracy of spiral CT in detection and assessment of post-intubation tracheal stenosis, length of stenosis was accurate in 14 (87%) of the 16 stenotic segments detected by CT and in 11 (73%) of the 15 segments detected by bronchoscopy [19]. In another study, rigid bronchoscopy was superior than flexible bronchoscopy and CT scan in evaluation of length of stenosis [20]. In our experience, all patients had CT scan which was used to grade degree of stenosis and determine the length. Rigid bronchoscopy is available only in one of our centers, hence, it was not used for patients treated at the other three centers.

The management armamentarium of tracheal stenosis has been expanding to include bronchoscopic dilation, neodymium-yttrium aluminum garnet (Nd-YAG) laser, photo-dissection or electro-knife, stent placement, cryotherapy, intralesional steroid injection, and mitomycin application in addition to surgical resection, tracheoplasty and tracheostomy [12]. In our institution, only tracheostomy, BD and tracheal REEA area available, and it would be our assumption that it is the same among most establishments within the developing countries. Selection of treatment options depends on availability of resources and expertise, patient condition as well as severity and length of stenosis. The gold standard treatment has been resection and anastomosis [7]. In one large series of surgically managed patients, good or satisfactory outcome was achieved in 96% [4]. However, lesser success rates of 61.5%(for multilevel stenosis) and 68.2% have also been reported [9, 21]. In our series, 41 (89.1%) had good or satisfactory outcome. As our experience with such procedure is only over last ten years, we expect better outcomes in the future. Similarly, the final outcome of the patients treated with BD alone, single session and repeated, was 75% although with a statistically significant higher rates of recurrence than the surgical group. The overall crossover from BD to tracheal REEA was 25% and when crossover was allowed, the rate of satisfactory outcome for the patients initially treated with BD becomes 92.6% which is not significantly different from the primary tracheal REEA group. In addition, the only factor that increased rate of failure for both BD and tracheal REEA was the length of TS. BD should be reserved for patients with shorted length and less narrowed stenosis, and if patients were to be initiated on BD and failed, earlier transition to surgery may help reduce further recurrences.

Different treatment algorithms have been reported in the literature. However, the practice is variable depending on availability of resources and expertise. Simple stenosis (less than 1 cm in length), granulomas and weblike stenosis are recommended to be treated with endoscopic options: dilation, laser, cryotherapy, or stents. Complex and > 1 cm long stenosis are generally offered surgical treatment ( [22]). Patients with complex stenosis who are not fit for surgery are also managed with endoscopic options ( [23]). However, these algorithms cannot be strictly followed in low resource settings. Surgery and permanent tracheostomy are still the main, or only options in Sub Saharan Africa ( [24]). In such settings, surgery should be offered for complex stenosis, and even simple stenosis if endoscopic options are not available.

The overall rate of satisfactory patient reported outcome of patients with TS managed either with BD or tracheal REEA was high. A quarter of patients initially treated with BD crossed over to the REEA treatment modality while only 6% required BD after REEA. The only factor that increased the rate of recurrence was the length of the stenosis. In our patient series, patients with narrower TS were assigned to REEA and had a good outcome, and patients treated with BD with a longer TS had a higher recurrence so we recommend that severe stenosis cases could benefit from direct REEA. Nonetheless, a prospective matched study is required to validate our findings.

## Strengths and limitations

This study provides relatively large cohort of patients with tracheal stenosis from limited resource setting. Patients were properly followed to observe for recurrences and management of recurrence. It reflects that even with lack of treatment modalities like laser ablation, stent placement, or routine availability of bronchoscope, good outcome can be achieved.

This study has several limitations. We could not follow treatment algorithms that typically can apply for well-developed set ups [22]. Because the only center for surgical or bronchoscopic treatment of our patients is found only in the capital city, Addis Ababa, many patients were placed on initial tracheostomy, while initial definitive treatment could have been done in developed set ups. Patients far away from the center took longer times. Some patients’ outcome could have been altered had they got definitive treatment early.

## Conclusion and recommendations

Tracheal stenosis is a potentially life threatening condition, that arises mostly because of previous intubation. Patients present with symptoms of upper airway obstruction. Surgical resection and anastomosis offers best outcome, however, for short segment and less severe stenosis, recurrence after surgery, or multilevel stenosis, bronchoscopic dilation offers good outcome too. In settings where the expertise or equipment for dilation are not available, tracheostomy should be done for severe stenosis before referral. Further studies should determine indications for each type of treatment. Guidelines emerging from published studies should develop standard treatment algorithm that takes resource limited settings into consideration.

## Data Availability

Data used for this study can be accessed upon request to the corresponding author.

## Abbreviations

ARDS: Acute Respiratory Distress Syndrome
BD: Bronchoscopic Dilation
GBS: Guillain-Barré syndrome
CT: Computed Tomography
HIV: Human Immunodeficiency Virus
PITS: Post intubation tracheal stenosis
PT: Primary Tracheostomy
REEA: Resection and End to End Anastomosis
TS: Tracheal stenosis
